# In This Issue

**DOI:** 10.1002/(SICI)1097-0061(200004)17:1<V::AID-YEA13>3.0.CO;2-C

**Published:** 2000


**Yeast**
*Yeast* 2000; **17**: v–vi.

### In this Issue

In this first issue of *Comparative and Functional Genomics* we have three commissioned reviews and one research article. In addition to these are the first of a set of features that we plan to have in every issue of CFG. These are a Featured Organism article, an Interview, a Website Review and a review of an International Meeting.

### Pharmacogenomics

There has been much attention paid in the media recently to the application of genomics, and in particular the sequence of the human genome, to medicine. The MRC and the Wellcome Trust recently announced that they are preparing to set up the UK Population Biomedical Collection, a database of medical records and lifestyle profiles combined with a bank of DNA samples of half a million UK residents aged between 45 and 65. The media interest has also been fuelled by the release of the near-complete sequence of human chromosome 22 (Dunham *et al.*, 1999). A consortium of pharmaceutical companies has come together to undertake a genome-wide single nucleotide polymorphism (SNP) mapping project in which they aim to type some 300 000 human SNPs. Ruth March's review provides a comprehensive picture of the results obtained so far in the arena of genetic studies of the variation in responses to known drugs within sample populations. This section of pharmacogenomics research will benefit greatly from the results of the SNP project and from the results of all the human genome sequencing projects. It is hoped that this work will one day lead to more cost-effective drug selection and tailored medicine.

### Insights into plant genome evolution

The recent release of the complete sequences for chromosomes 2 and 4 of the thale cress, *Arabidopsis thaliana* (Lin *et al.*, 1999; Mayer *et al.*, 1999), caused much excitement, particularly amongst plant genome researchers. Ian Bancroft discusses the implications of these results in the study of the functional and structural evolution of plant genomes in his review.

### Comparative mapping display methods

Comparative mapping is a valuable tool for within- and between-species studies, in the construction of genetic and physical maps and in the study of genome evolution. A crucial part of comparative mapping is the clear and meaningful display of results. Jo Dicks reviews the currently available methods for the graphical display of comparative maps and discusses the efforts under way to improve upon these systems.

### *Fugu* and human synteny study

The research paper of McLysaght *et al.* details the results of a comparative study of the *Fugu* and human genomes. They have investigated the level of compaction of the *Fugu* genome as compared to the human genome and assessed whether this varies with base composition. They have also used a computational model to estimate the number of chromosomal rearrangements that may have occurred since the *Fugu* and human lineages diverged. They also estimated how much synteny remains between *Fugu* and man, this allows an evaluation of how useful *Fugu* will be as a model genome.

### Featured Organism: the nematode worm, *C. elegans*

The Featured Organism article in this issue is the nematode worm, *Caenorhabditis elegans.* The feature article gives background information on this important model organism, which boasts the first completely sequenced animal genome. There are details of the work currently under way to assign function to the many genes predicted from the sequence and a list of web sites providing further information on *C. elegans*. The article concludes with a discussion of the future direction of

Copyright © 2000 John Wiley & Sons, Ltd.

nematode functional and comparative genomics, with comments from three prominent worm genomics researchers. This article was written to coincide with the interview with Alan Coulson and Patty Kuwabara, who are members of the nematode functional genomics team at the Sanger Centre. They are coordinating the gene knockout project for the worm, which aims to provide worms mutated in chosen genes for phenotypic analysis by the worm community. The interview covers the gene knockout project and the other approaches currently being applied to nematode functional genomics.

### Website Review: KEGG

The Kyoto Encyclopedia of Genes and Genomes is the subject of this issue's Website Review. This highly organized web site provides a comprehensive collection of metabolic and regulatory pathways and the genes involved in these that have been identified in a wide range of organisms. There are also links to completely sequenced genomes and chromosomes and to functionally categorized lists of the genes predicted from these sequences. There are genome map and comparative map viewing tools and extensive search facilities available, making this site a good starting point for finding information on a pathway or a gene in many organisms.

### Meeting Highlights: PAG VIII and AMG I

The Meeting Highlights in this issue covers Plant and Animal Genomes VIII and Agricultural Microbe Genomes I, which were held back-to-back in San Diego, 9–14 January 2000. An enormous amount of comparative mapping of animal and plant genomes is under way. However, functional genomics studies were more prevalent in the plant community. The use of membrane-based cDNA microarray technology is seen as the way forward for the study of those plants whose genomes are too large and too repeat-rich to be suitable for sequencing. Databases and technological advances of interest to all genome projects were also reported and demonstrated at PAG VIII. AMGI hosted presentations on studies of plant and animal pathogens and included the announcement of the first complete genome sequence of a plant pathogen, *Xylella fastidiosa*. A common long-term aim of these studies is the identification of virulence-related genes and in some cases, impressive progress has been made towards this goal. Several projects are utilizing comparisons to non-pathogenic (or avirulent) strains and functional analyses, such as expression analysis using microarrays, are becoming more widely used.

*Comparative and Functional Genomics* is a cross-organism journal, and we have tried to reflect that in the content of this first issue, whilst commissioning topical reviews. It is our aim, whenever possible, to provide a balanced selection of articles in each future issue.

### References

Dunham I, Hunt AR, Collins JE, *et al.* 1999. The DNA sequence of human chromosome 22. *Nature* **402**: 489–495.

Lin X, Kaul S, Rounsley S, *et al.* 1999. Sequence and analysis of chromosome 2 of the plant *Arabidopsis thaliana*. *Nature* **402**: 761–768.

Mayer K, Schüller C, Wambutt R, *et al.* 1999. Sequence and analysis of chromosome 4 of the plant *Arabidopsis thaliana*. *Nature* **402**: 769–777.

Copyright © 2000 John Wiley & Sons, Ltd.
